# Defining and reporting exercise intensity in interventions for older adults: a modified Delphi process

**DOI:** 10.1186/s11556-024-00337-8

**Published:** 2024-02-02

**Authors:** Bettina Wollesen, Mona Herden, Nicola Lamberti, Christoforos D. Giannaki

**Affiliations:** 1 Institute of Human Movement Science, University of Hamburg, Hamburg, Germany; 2 Department of Neuroscience and Rehabilitation, University of Ferrara, Ferrara, Italy; 3 Department of Life Sciences, School of Life and Health Sciences, University of Nicosia, Nicosia, Cyprus

**Keywords:** Exercise intensity, Older adults, Expert rating, Delphi process

## Abstract

**Background:**

Many exercise studies, including older adults, do not report all relevant exercise characteristics. Especially the description of exercise intensity is missing and mostly not controlled. This leads to difficulties in interpreting study results and summarizing the evidence in systematic reviews or meta-analyses. Therefore, the aim of the present Delphi study was to gain recommendations about the categorization of exercise intensity and for the conducting and reporting of characteristics in future intervention studies with older adults by experts in exercise science and physiology.

**Methods:**

Two hundred ninety-seven international interdisciplinary participants from an EU COST action were invited to participate in three rounds of online questionnaires in April/May 2023. Up to *N* = 93 experts participated in each round. Round 1 included open-ended questions to solicit possible recommendations and categorizations for light, moderate, vigorous, and high intensity. In round 2, the experts rated their agreement using Likert scales (1–10) on the revealed categories and recommendations. Clusters with a higher average rating of M = 8.0 were summarized into round 3. In the final round, the results were presented for a final rating of agreement (based on a simple majority > 50%).

**Results:**

In round 1 a total of 416 qualitative statements were provided from thirteen questions. From round 1 to round 3, a total of 38 items were excluded, with 205 items retained for the final consensus. In round three 37 participants completed the whole questionnaire. The experts showed overall agreement on the final categorizations with 6.7 to 8.8 out of 10 points on the Likert scale. They also showed broad consensus on the relevance of reporting exercise intensity and the recommendations for future conducting and reporting of study results. However, exercise types such as yoga, balance, and coordination training led to conflicting results for categorization into light or moderate.

**Discussion and implications:**

The results of the current survey can be used to classify the intensity of exercise and suggest a practical approach that can be adopted by the scientific community and applied when conducting systematic reviews and meta-analysis articles when vital and objective information regarding exercise intensity is lacking from the original article.

## Highlights

- Experts highlight the importance of reporting exercise intensity for individualization and participant safety

- International interdisciplinary participants with expertise in exercise gained a common understanding of the categorization of exercise intensity for future intervention studies with older adults.

- Conflicting results for exercise types such as yoga, balance, and coordination training present categorization challenges

## Introduction

Exercise is considered an effective, nonpharmacological approach in terms of improving various health and quality of life-related parameters in older adults [1]. A large body of evidence supports the beneficial effect of different forms of exercise in a cascade of physiological, mental, cognitive, and social health outcomes, and many older individuals are currently engaging in organized exercise training programs worldwide [2–5]. Thus, it is not surprising that older adults who systematically participate in exercise programs have a reduced risk for the development or aggravation of an existing chronic disease, including cardiovascular diseases [6], diabetes [7], and dementia [8], while they may maintain their independent living ability and good levels of physical and cognitive functionality [1].

It seems that exercise forms are superior in terms of the level of adaptations compared to physical activity forms such as work-related physical activity. For instance, work-related physical activity failed to reduce the risk of metabolic syndrome [9]. Similarly, Schmidt et al. (2017) showed that work-related physical activity did not significantly affect health and physical fitness, while habitual physical activity was less effective than sports-related activities (higher intensity activities) [10].

Concerning benefits that can be derived from different forms of physical activity, it must be noted that physical exercise and physical training are more specific in terms of structure and planning. Physical exercise (PE) is called a subset of physical activity that is planned, structured, and repetitive and should be distinguished on temporal characteristics into (i) *acute physical exercise* (single bout) and *chronic physical exercise* (repeated bouts of acute exercise) [1, 11, 12]. Chronic physical exercise conducted regularly in a planned, structured, and purposive manner with the objective of increasing (or maintaining) individual capabilities in one or multiple fitness dimensions is also called physical training [12].

Current physical activity guidelines and homogenous exercise prescriptions across health organizations (i.e., WHO) often fail to improve physical fitness and health parameters. On the other hand, personalized exercise prescription programs are reported to be more effective at enhancing such parameters [13].

Many forms of exercise have been shown to be suitable for older adults, including aerobic, resistance, flexibility, and balance training, swimming, Tai-Chi, combined hybrid forms (e.g., aerobic and resistance exercises), and others. However, their characteristics are highly diverse [14]. Moreover, in recent decades, new forms of exercise training have appeared, in which the most critical characteristic is high exercise intensity (e.g., high-intensity interval training, high-intensity functional training). These forms of exercise are currently effective and very popular and can be applied in various settings, including gyms, nursing homes, and hospitals [15, 16].

Therefore, exercise regimens, especially if they are conducted for older adults, should include some relevant training principles to secure potential adaptations. There is much evidence that goal-directed PE should integrate a certain individualization (e.g., to consider the individual’s health and fitness level), should be specific in triggering the necessary adaptations of a specific health condition (e.g., endurance training to improve the cardio-respiratory system) and must gain a certain overload to be effective [17].

Exercise programs for older adults should be individualized and tailored, and they need to account for the individual’s needs, health status, fitness levels, and willingness to exercise to favor optimal adaptations. Moreover, to prescribe and monitor effective and safe exercise training programs, exercise professionals use principles such as frequency, intensity, time, and type (F.I.T.T.) that act as an essential component in evidence-based medicine [17]. Thus, it is not surprising that the intensity of exercise was used as a moderator in meta-analyses related to the effects of exercise on various health-related parameters [18].

When prescribing exercise in older people, it is essential to tailor it to each individual, taking into consideration their specific goals and needs. This includes personalizing the modality, frequency, duration, and intensity of physical activity. Intensity is one of the cornerstones of exercise programs and a continuous topic of interest and debate among exercise scientists [19, 20]. Practically, intensity is defined by the amount of energy required for the performance of the physical activity per unit and time [21].

Exercise intensity is strongly related to external workload, which in turn affects the internal metabolic and cardiovascular stress in the human body and thus the level and the type of adaptation [20]. For instance, a recent meta-analysis reported that exercise intensity had a significant moderating effect in strength or resistance training exercise studies and yoga or tai chi trials [18]. In particular, an increased exercise intensity of 10% resulted in a significantly increased antidepressant effect [18]. Similarly, exercise intensity appears to moderate the effect of cardiorespiratory benefits induced by aerobic exercise. For instance, moderate-to-vigorous-intensity and vigorous-intensity exercise interventions were more effective in terms of improving relative VO2peak compared to low-intensity exercise in patients with cardiovascular diseases [22]. When it comes to resistance training, studies have shown that gradually increasing the intensity of the exercises to levels between 70 and 80% of an individual's one-repetition maximum (1RM) results in greater improvements in strength compared to training with lighter weights (less than 50% of 1RM) or moderate weights (less than 70% of 1RM) [23]. Moreover, metabolic stress induced by exercise (also affected by exercise intensity), prescribed relative to the percentage of VO_2_max (i.e., exercise above or below the anaerobic threshold), could partly explain the considerable interindividual variation in response reported following training programs using this method of exercise prescription [24]. As a consequence, exercise intensity should be carefully dosed when prescribing personalized exercise training programs in older adults, especially in those with chronic diseases [25]. Moreover, the progression (including training loads and dosage) of the exercise training program considering the issue of exercise intensity is an even more complicated issue in exercise training studies in older adults. Especially for older adults with chronic diseases and comorbidities, precise exercise prescription would be essential for training-induced adaptations but also for the safety of the participants [25].

Some published and popular recommendations that exercise scientists adopted in terms of exercise prescription were provided by the American College of Sports Medicine [26] and previous position papers [27, 28]. When we refer to other forms of exercise, such as balance, multimodal exercise, mind–body exercise forms, Pilates, Tai-Chi, and others, the prescription of exercise intensity is complicated. Moreover, these exercise forms have many subforms with different exercise characteristics, including exercise intensity, and can lead to diverse adaptations. For example, in a recent study from da Silva Almeida et al. (2021), it was found that a nontraditional approach to Pilates, with multiple sets, high repetitions, and shorter rest intervals, resulted in more significant energy expenditure and higher heart rates compared to a traditional Pilates session [29]. As a result, many studies do not even mention how exercise intensity was prescribed [29], and the outcomes may have been affected or even did not show benefits after the exercise training intervention. In addition, there is disagreement regarding the different methods that should be used regarding the most efficient and safe exercise training prescription for older adults.

In summary, within the current literature, there are some deficits in reporting exercise characteristics. This leads to insufficient information about the potential mechanisms of the exercise intervention. Especially within a meta-analysis to describe the effect sizes of beneficial exercise programs, current quality assessments for RCTs do not ask for or require a clear exercise description. Moreover, this area of research is an interdisciplinary field including sports and exercise scientists, clinical researchers, gerontologists, physiotherapists, public health researchers and human movement scientists. Consequently, a comprehensive understanding of exercise characteristics might be missing; for instance, many clinicians, especially primary care staff, experience difficulties in prescribing exercise in the presence of different concomitant chronic diseases due to a lack of expertise, knowledge, and skills [30].

Within the realm of health science, Delphi processes function as a methodical and organized approach to achieving consensus among a group of experts regarding a specific topic or question. This methodology proves invaluable for accessing the combined expertise of professionals, facilitating well-informed decision-making, and dealing with intricate issues requiring unanimous agreement [31]. Its efficacy is especially pronounced in scenarios where in-person discussions are impractical, or when the amalgamation of diverse viewpoints is necessary to inform the development of policies, guidelines, or research priorities.

A standardized checklist consisting of 16 items was formulated in a previous Delphi study to ensure accurate reporting of exercise programs in clinical trials. However, it should be noted that this checklist was not specifically tailored for older adults [31]. Moreover, this checklist was provided to secure high-quality reporting of future RCTs. Specific recommendations on how to deal with missing reporting of intensity for ongoing meta-analysis was missing in this Delphi process [32].

Therefore, this Delphi survey aimed to collect the opinions of experts with different backgrounds, especially in exercise science of a European-funded COST action program, “PhysAgeNet”, about exercise intensity in older adults and use their judgment to define and classify exercise intensity.

The main research questions are:

- Why should training intensity be reported according to the expert's opinion?
  - What are the main reasons for reporting exercise intensity?
  - Which benefits can be gained from reporting exercise intensity?

- Which exercise would the experts rate as light, moderate, vigorous, and high?
- Which recommendation for the description of exercise characteristics for a future gold standard can be gained by the expert’s rating?

## Materials and methods

### Study design

The Delphi survey comprising three rounds of open (first and second round) and closed questions (second and third round) was conducted following the recommendations of Trevelyan and Robinson (2015) [33]. Informed consent was confirmed by all participants before completing each questionnaire in accordance with the Declaration of Helsinki (2018). Data collection took place between April and June 2023.

### Participants

According to recommendations in the literature [33, 34], a minimum sample size of at least ten participants is required. A maximum was not determined, as we aimed to integrate as much expertise as possible.

Inclusion criteria solely comprised expertise in a field relevant to exercise science or exercise physiology in older adults, such as medicine or human movement science. Experts to participate were searched within the EU-COST network PhysAgeNet. The main aim of PhysAgeNet is to establish a sustainable network that will foster evidence-based research and the practice of physical activity in older adults and will enhance the integration of innovative ICT solutions based on open data consolidated research information to promote health and reduce the burden of inactivity in older adults (https://physagenet.eu/).

A total of *N* = 297 international interdisciplinary experts from 43 countries were invited to participate via email.

### Study flow

**Fig. 1:**
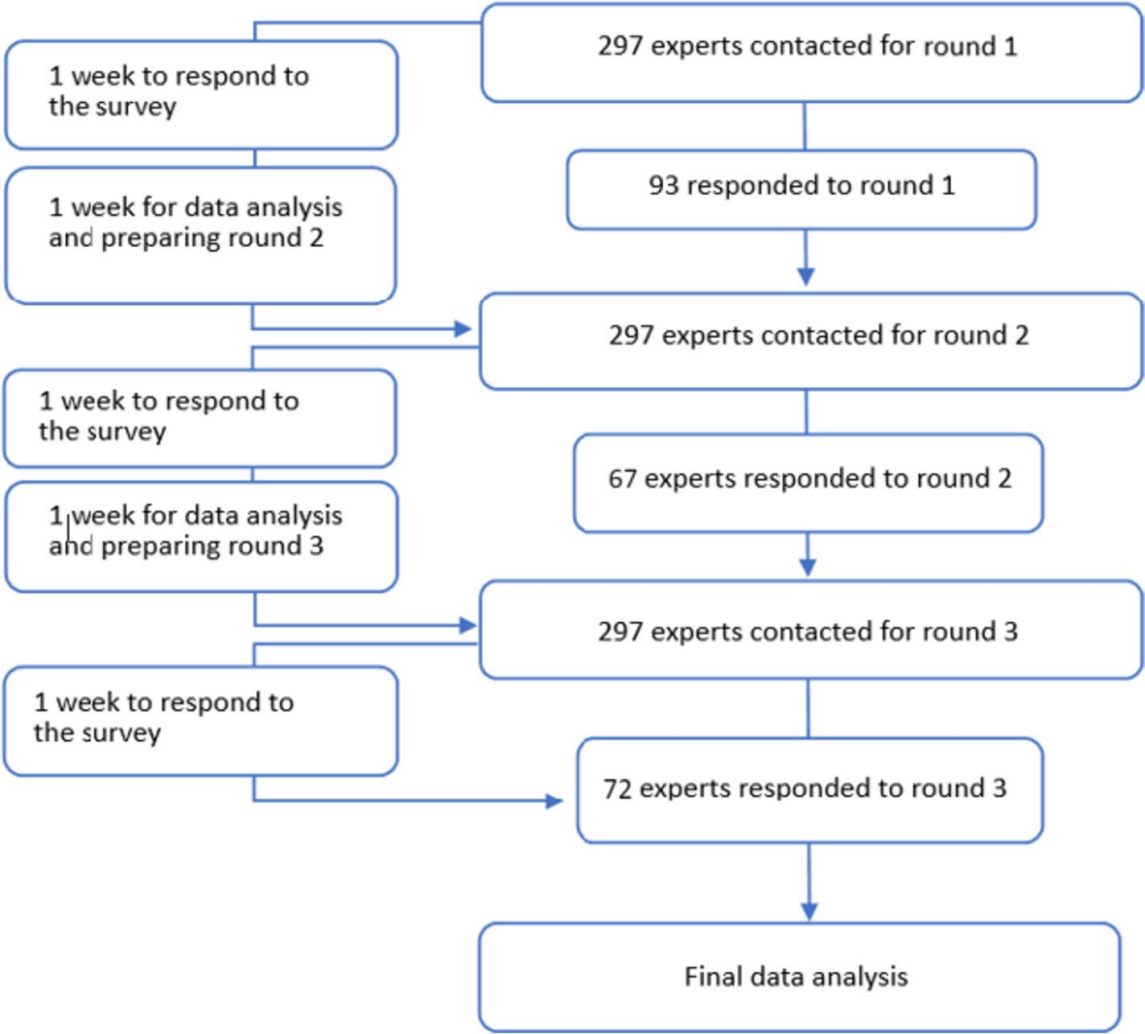
Study flow and participation

Potential participants of the COST action received an email with a letter of invitation including the content and purpose of the study, as well as rules of participation and the link to access the respective questionnaire. Each round of the Delphi survey comprised an online questionnaire created in the software “LimeSurvey” (LimeSurvey GmbH, Hamburg, Germany). The survey tool, LimeSurvey, and the questionnaire's contents were tested and approved by volunteers a priori. The three rounds were conducted at intervals of two weeks. Each questionnaire was supposed to be completed within one week. Afterwards, the results were analyzed and incorporated in the next round within one week (Fig. 1).

Participants who did not complete the questionnaire within four days received a reminder via email to enquire about the intention to participate.

### Questionnaires

Following the onset of the Delphi process, the current predominant comprehension of exercise intensity was summarized according to actual guidelines and recommendations (e.g., [27, 28]). Afterwards, the questions for the first round were developed. The questionnaire was piloted with a small group of experts from exercise physiology and exercise science (*n* = 9) of the EU network PhysAgeNet.

### First round of the Delphi process

The first Delphi questions included four blocks (see the supplemental material for further information) of open questions for qualitative analysis:

1. Comprehension of reporting exercise intensity for older adults (> 60 years following WHO definition; WHO, 2002 [34]) via open questions.
2. Indication of relevant exercises that are rated as light, moderate, vigorous, and high intensity.
3. Recommendations to derive or calculate exercise intensity if no objective or subjective measurement is available as well as to describe the intensity of hybrid or multicomponent programs.
4. Recommendations to improve reporting quality of exercise intervention studies in older adults (> 60 years).

After the first round, two additional rounds were executed.

### Second round of the Delphi process

In the second Delphi round, following the separation of the exercises according to intensity levels, the experts were asked to rate the keywords of light-intensity exercise definitions retrieved from the first Delphi round on a 1–10 Likert scale. Clusters with a higher average rating of M = 8.0 were further summarized into the third round.

Here, qualitative answers were also possible if the experts missed any aspects. All items that gained a simple majority (> 50%) and clusters with a higher average rating of M = 8.0 were further summarized into the third round for the final consensus.

### Third round of the Delphi process

The final third round was conducted to gain an overall agreement on the recommendations on how to report exercise intensity if no objective or subjective measurement was available.

In addition to basing each round’s content on the previous round’s results to reach a consensus, the ratings from round two offered free-text comments to specify and clarify ratings or disagreements with the given answers.

The activities were categorized by the expert consensus based on the answers given by the participants. The compendium of physical activities [27] was helpful due to its well-structured display of METs for different physical activities (cf. Table 4). These METs were utilized in certain inquiries within the Delphi survey.

All experts were invited to be mentioned in the publication (see acknowledgments).

The first round took approximately 20 min to complete the questionnaire, and the second and third rounds could have been finished in 10 to 15 min.

### Data processing and analysis

According to the nature of a Delphi procedure, qualitative (rounds 1 and 2) and quantitative (rounds 2 and 3) results were extracted from the answers to the questionnaires. Qualitative results were analyzed according to Mayring (2010) [35] by categorizing the content to transfer it into closed questions. This categorization was done by two of the authors independently (MH and BW). The categorization was presented to the two other authors (CG and NL). Any disagreement between MH and BW was supervised and moderated by CG and NL. The quantitative results were descriptively summarized. Participants were categorized by their profession: sports and exercise sciences, physiotherapy and rehabilitation, medicine, and others (nursing science, health care professionals).

The process of categorization of exercise intensity for older adults was made in four steps:

1. Identifying the types of exercise that were named by the experts for the categories light, moderate, vigorous, or high (round 1).
2. Summarizing the exercise types and categorization aspects in closed questions for the rating of the experts via Likert scales (round 2). Clusters with a higher average rating of M = 8.0 were further summarized into the third round.
3. Delete answers that did not have more than 50% agreement by the experts to present a simple majority (round 3).
4. Summary of additional recommendations for combined or multicomponent exercises as well as balance and coordination training (round 2 and 3).

## Results

### Sample characteristics

The response rate for the first round was 33.7%. The rate of complete answers from total participation in round 1 was 35.5%. The distribution of incomplete and complete answers are displayed next to the total participation per round and the distribution of professions among the participants are presented in Table 1.

**Table 1:** Participants overview

Involvement	Participation		
	Incomplete	Complete	Total
Round 1	60	33	93
Round 2	34	33	67
Round 3	35	37	72
Distribution of Professions^a^			
Sports and Exercise Sciences	22	15	22
Physiotherapy and Rehabilitation	16	7	16
Medicine	13	7	13
Others	7	4	7
No profession available	2	0	33

^a^Distribution of professions in round 1: Detailed overview of profession distribution of complete participant s see appendix (Table 8)

In the first round, sixty of a total of ninety-three participants included the confirmation of privacy policy; thus, valid answers were distributed over the expert groups: sports and exercise sciences (*n* = 22), physiotherapy and rehabilitation (*n* = 16), medicine (*n* = 13), and others (*n* = 7). A total number of *n* = 33 participants refused to mention their profession. All given answers from all three stages were included in the analysis.

### Relevance of reporting exercise intensity in exercise studies with older adults

In the first round of the Delphi process a total of 416 qualitative statements were provided from thirteen questions. These statements were deduced to a total of 243 items in the second round. Clusters with a higher average rating of M = 8.0 were further summarized into the third round, thus 38 items were excluded and 205 items were retained for the following summary of reasons to consider and report exercise intensity in exercise studies with older adults (cf. Table 2):

**Table 2:** Agreement of the reasons and benefits for reporting and analyzing exercise intensity in interventions for older adults

Domain	Main reasons for reporting and analyzing exercise intensity (% of experts ratings ≥ 7)	Average Rating (M ± SD)
Deductions	It is an important term of exercise prescription. (98%)	9.4 ± **1.0**
	It helps to design loads and progression of an exercise program. (93%)	9.0 ± **1.5**
	It is a way of measuring dosage. (88%)	8.6 ± **1.7**
	It helps to facilitate the benefits of exercise. (86%)	8.4 ± **1.6**
	It provides objective data on the effectiveness of the exercise program. (83%)	8.4 ± **1.7**
	It increases the quality of knowledge regarding the area of exercise benefits in older adults. (81%)	8.4 ± **1.9**
Individualization	It helps to design loads and progression of an exercise program. (93%)	9.0 ± **1.5**
	It helps to control physical adaptations. (91%)	8.6 ± **1.5**
	It is a critical component especially in age- related decreases in physical performance. (83%)	8.4 ± **1.9**
	The same criteria do not suit all ages and therefore should be defined. (78%)	7.7 ± **2.4**
Safety	It helps to prevent any harm or adverse events. (76%)	8.0 ± **2.0**

*Abbreviations: M* Means, *SD* Standard deviation

### Categorization of exercise intensity

In Table 3, the suggestions of the first round by all participants for the four categories of exercise intensity can be found:

**Table 3:** First Delphi round: Categories captured from the experts voting for the definition process of light-, moderate-, vigorous- and high-intensity exercises

Light	Moderate	Vigorous	High
**Walking** (*n* = 19) Physiotherapy and Rehabilitation (*n* = 6), Sports and Exercise Sciences (*n* = 5), Medicine (*n* = 6), Other (*n* = 2) **Balance** (*n* = 6) Sports and Exercise Sciences (*n* = 4), Physiotherapy and Rehabilitation (*n* = 1), Medicine (*n* = 1) **Exercise with MET less < 3.1** according to Ainsworth’s compendium of physical activities (*n* = 5) Sports and Exercise Sciences (*n* = 3), Physiotherapy and Rehabilitation (*n* = 1), Medicine (*n* = 1) **Yoga** (*n* = 5) Sports and Exercise Sciences (*n* = 4), Medicine (*n* = 1), Other (*n* = 1) **Stretching** (*n* = 5) Medicine (*n* = 2), Physiotherapy and Rehabilitation (*n* = 1), Sports and Exercise Sciences (*n* = 1), Other (*n* = 1) **Coordination** (*n* = 3) Sports and Exercise Sciences (*n* = 2), Medicine (*n* = 1) **Functional and daily activities** (*n* = 3) Sports and Exercise Sciences (*n* = 2), Physiotherapy and Rehabilitation (*n* = 1) **Exercises that allow easy talking** (*n* = 3) Physiotherapy and Rehabilitation (*n* = 1), Sports and Exercise Sciences (*n* = 1), Medicine (*n* = 1) **Tai-Chi** (*n* = 3) Sports and Exercise Sciences **Qigong** (*n* = 2) Sports and Exercise Sciences **Running** (*n* = 2) Physiology and Rehabilitation (*n* = 1), Sports and Exercise Sciences (*n* = 1) **Pilates** (*n* = 2) Physiotherapy and Rehabilitation (*n* = 1), Sports and Exercise Sciences (*n* = 1) **Cycling** (*n* = 1) Medicine **Slow Ballroom dancing** (*n* = 1) Sports and Exercise Sciences **Feldenkrais** (*n* = 1) Sports and Exercise Sciences **Taijiquan** (*n* = 1) Sports and Exercise Sciences	**Walking/**nordic walking/group walking (*n* = 15) Sports and Exercise Sciences (*n* = 5), Other (*n* = 4), Medicine (*n* = 3), Physiotherapy and Rehabilitation (*n* = 2) **Cycling/slow cycling** (*n* = 5) Sports and Exercise Sciences (*n* = 2), Medicine (*n* = 2), Physiotherapy and Rehabilitation (*n* = 1) **Exercise rated with MET 3.2–4.7/MET 3–6** (*n* = 6) Sports and Exercise Sciences (*n* = 4), Physiotherapy and Rehabilitation (*n* = 1), Medicine (*n* = 1) **Dance/**dance exercise/fast ball-room dancing (*n* = 5) Sports and Exercise Sciences (*n* = 3), Physiotherapy and Rehabilitation (*n* = 1), Medicine (*n* = 1) **Bodyweight exercise** (*n* = 5) Sports and Exercise Sciences (*n* = 2), Medicine (*n* = 2), Physiotherapy and Rehabilitation (*n* = 1) **Able to sustain steady conversation** (*n* = 4; Sports and Exercise Sciences (*n* = 3), Other (*n* = 1) **Water aerobics/aerobics** (*n* = 3) Physiotherapy and Rehabilitation (*n* = 1), Sports and Exercise Sciences (*n* = 1), Other (*n* = 1) **Pilates/Yoga** (*n* = 3) Medicine (*n* = 2), Sports and Exercise Sciences (*n* = 1) **Submaximal exercise** 60–70%/55–60% Hfmax (*n* = 3) Physiotherapy and Rehabilitation (*n* = 1), Sports and Exercise Sciences (*n* = 1), Medicine (*n* = 1) **Slow jogging/jogging** (*n* = 2) Physiotherapy and Rehabilitation (*n* = 1), Sports and Exercise Sciences (*n* = 1) **Stair climbing** (*n* = 2) Physiotherapy and Rehabilitation **Functional training/Calisthenics** (*n* = 1) Sports and Exercise Sciences **Moderate intensity interval training** (*n* = 1) Other **Lifting weights** (*n* = 1) Physiotherapy and Rehabilitation **Golf** (*n* = 1) Sports and Exercise Sciences **Exergaming** (*n* = 1) Sports and Exercise Sciences **Housework** (*n *= 1) Other **Gardening** (*n* = 1) Other	**Running/**treadmill running/uphill jogging (*n* = 14) Sports and Exercise Sciences (*n* = 5), Physiotherapy and Rehabilitation (*n* = 4), Medicine (*n* = 3), Other (*n* = 2) **Exercise rated with METs 6–9** (*n* = 5) Sports and Exercise Sciences (*n* = 4), Physiotherapy and Rehabilitation (*n* = 1) **Weight training** (*n* = 4) Physiology and Rehabilitation (*n* = 2), Sports and Exercise Sciences (*n* = 1), Medicine (*n* = 2) **Swimming** (*n* = 4) Physiotherapy and Rehabilitation (*n* = 3), Other (*n* = 1) **Able to sustain few words/**difficulties to speak/conversation generally cannot be maintained uninterrupted up to 30 min (*n* = 5) Sports and Exercise Sciences (*n* = 2), Medicine (*n* = 1), Other (*n* = 2) **Power walking** (*n* = 3) Medicine (*n* = 2), Physiology and Rehabilitation (*n* = 1)) **Cycling** (*n* = 3) Sports and Exercise Sciences (*n* = 2), Medicine (*n* = 1) **Climbing stairs** (*n* = 2) Physiology and Rehabilitation (*n* = 1), Medicine (*n* = 1) ** < 70% of HRmax** (*n* = 2) Physiotherapy and Rehabilitation (*n* = 1), Sports and Exercise Sciences (*n* = 1) **Aerobic dancing** (*n* = 1) Sports and Exercise Sciences **METs < 4.8** (*n* = 1; Sports and Exercise Sciences **Exercise training following the FITT principle** (*n* = 1) Sports and Exercise Sciences **Sport games** (Soccer, basketball, volleyball etc.) (*n* = 1) Other **Climbing** (*n* = 1) Other **High intensity interval training** (*n* = 1) Other **Skiing** (*n* = 1) Other	**Running** (*n* = 4) Physiotherapy and Rehabilitation (*n* = 2), Medicine (*n* = 1), Other (*n* = 1) **Same as vigorous intensity** (*n* = 4) Sports and Exercise Sciences (*n* = 3), Physiotherapy and Rehabilitation (*n* = 1) **Cycling** (*n* = 3) Sports and Exercise Sciences (*n* = 1), Physiotherapy and Rehabilitation (*n* = 1), Medicine (*n* = 1) **More than 80% HRmax** (*n* = 2) Physiotherapy and Rehabilitation (*n* = 1), Sports and Exercise Sciences (*n* = 1) **High intensity interval training** (*n* = 2) Sports and Exercise Sciences (*n* = 1); Other (*n* = 1) **Rope jumping** (*n* = 2) Sports and Exercise Sciences (*n* = 2), Medicine (*n* = 1) **Squash** (*n* = 1) Sports and Exercise Sciences) **Sport games** (Soccer, basketball, volleyball) (*n* = 1) Other **Cross country skiing** (*n* = 1) Other **Long distance swimming** (*n* = 1) Other **Climbing** (*n* = 1) Other

In the second Delphi round, the experts were asked to rate the keywords of light-intensity exercise definitions retrieved from the first Delphi round on a 1–10 Likert scale. The finalized keywords for light-intensity exercises with average Likert-scale ratings > 7 were assessed for agreement in the third Delphi round. The same process was performed for the other categorizations. In total, thirteen questions with twenty rating items were given in the second Delphi round, with a total item count of *n* = 243 for the second Delphi round and a total item count of *n* = 205 for the third round. For moderate intensity, the items Lifting weights (6.5 ± 2.9), Golf (6.4 ± 2.7), Gardening (6.2 ± 2.9), and Housework (5.8 ± 2.8) were excluded because they did not gain the required rating of the experts. Within the vigorous section, the item workout with workout video was excluded. Moreover, the answers that did not directly follow the questions were excluded. These were exercises rated with MET 3.2—4.7, examples by the American Heart Association, 60–70% HRmax, 55–60% HRmax (7.7 ± 2.5) for moderate intensity and 70–74% HRmax (8.9 ± 1.5) and recommendations of the American Heart Association (8.2 ± 2.6) for vigorous intensity. Additionally, some experts stated that the intensity follows the individual fitness state, which was deleted here, because this answer did not match the question. However, these suggestions were kept for the overall recommendations.

The overall expert rating for the four exercise categories light, moderate, vigorous, and high can be found in Table 4.

**Table 4:** Categorization of relevant exercises that are rated as light, moderate, vigorous, and high intensity

Category	Description	Rating (Mean + SD)	Category	Description	Rating (Mean + SD)
**light**	Exercises that allows easy talking (89%)	8.8 ± **1.8**	**moderate**	Exercise that allows steady conversation (69%)	7.7 ± **2.4**
	Exercises rated with METs < 3 (82%)	8.4 ± **2.5**		Exercises rated with MET 3.1 -6 (89%)	8.4 ± **2.2**
	Stretching/Flexibility exercises (82%)	8.2 ± **2.4**		Nordic walking/uphill walking/group walking (78%)	7.7 ± **2.3**
	Daily life functional activity (82%)	8.2 ± **2.3**		Interval training (80%)	7.7 ± **2.4**
	Walking (< 4.8 km/h) without significantly increasing HR (79%)	8.1 ± **2.4**		Cycling/Slow cycling (78%)	7.6 ± **2.5**
	Walking (73%)	7.6 ± **3.0**		Water aerobics/Aerobic (72%)	7.6 ± **2.5**
	Balance Exercises (74%)	7.3 ± **2.8**		Dance (81%)	7.6 ± **2.3**
	Coordination Exercises (70%)	7.2 ± **2.6**		Pilates (76%)	7.4 ± **2.5**
	Slow ball-room dancing (61%)	7.1 ± **2.5**		Slow jogging (68%)	7.4 ± **2.8**
	Slow mind–body exercises like Tai-Chi, Qigong, Feldenkrais Yoga, Taijiquan, Pilates (65%)	7.0 ± **2.8**		Stair climbing (70%)	7.3 ± **2.5**
	Yoga (58%)	6.7 ± **3.0**		Exergaming (63%)	7.0 ± **2.5**
**vigorous**	Exercise allows sustain few words (81%)	7.9 ± **2.6**	**high**	Not able to talk a few words (72%)	7.6 ± **2.5**
	Exercises rated with MET 6–9 (84%)	8.4 ± **2.7**		METsas classified in literature (78%)	8.1 ± **2.6**
	Cannot recover breathing, cramping, tremors in movement (80%)	7.5 ± **3.1**		Cannot be sustained for more than 10 consecutive minutes (72%)	7.3 ± **2.8**
	Running/Treadmill running/uphill jogging (86%)	8.5 ± **2.4**		High intensity interval training (92%)	8.8 ± **2.2**
	Power walking (83%)	8.1 ± **2.5**		Training with high loads (close to 1-RM) (86%)	8.5 ± **2.3**
	Aerobic dancing (81%)	8.0 ± **2.4**		Running (80%)	7.7 ± **2.7**
	Sport games (soccer, basketball, volleyball) (75%)	7.8 ± **2.5**			
	Climbing (84%)	7.7 ± **2.8**			
	Skiing (80%)	7.6 ± **2.6**			
	Cycling (74%)	7.4 ± **2.4**			
	Weight training (72%)	7.4 ± **2.6**			
	Swimming (72%)	7.4 ± **2.2**			

*Abbreviations*: *HR* Heart rate, *RPE* Rating of perceived exertion, *METs* Metabolic equivalent of task

There was also a suggestion that high and vigorous intensity should be summarized as a unique category (89%, 8.4 ± 2.3).

The results of the experts´ suggestions on how to deal with combined or multicomponent exercises as well as for programs with a progression can be found in Table 5.

**Table 5:** Recommendations to derive or calculate exercise intensity if no objective or subjective measurement is available and to describe the intensity of hybrid or multicomponent programs

Calculation of multimodal or combined exercises	Average Rating (M ± SD)
Weighted average of the different exercise components (86%)	8.5 ± **2.1**
The different components of exercise should be treated separately (74%)	7.5 ± **2.4**
**Classification of exercise intensity for older adults for balance and coordination training**	
Looking at the exercise description (if available) and judging based on the pace of the movements and the strength requirements (71%)	7.9 ± **2.1**
Consider the categorization given by the authors (66%)	7.3 ± **2.6**
If the exercise includes dual tasking or also has a cognitive distraction or a secondary physical task while practicing a balance or coordination task is moderate (66%)	7.1 ± **2.7**
Consider these forms as ‘light’ intensity exercise (60%)	7.0 ± **2.6**
**Calculate intensity if there is a progression within the program for older adults**	
HR, RPE, METs, perceived exertion (91%)	8.7 ± **1.6**
% Delta Change from the original level of training (92%)	8.3 ± **1.9**
Giving the lowest and the highest value and the rate of progressivity (e.g., self-paced, % per week, etc.) (91%)	8.3 ± **1.7**
Use the mean value (71%)	7.9 ± **2.5**
Considering the exercise intensity that was sustained for a higher duration to be used as the exercise intensity for the training program (81%)	7.7 ± **2.2**

*Abbreviations*: *HR* Heart rate, *RPE* Rating of perceived exertion, *METs* Metabolic equivalent of task

For coordination exercises, there was also the suggestion that if the exercise includes stepping, it is moderate to vigorous (60%, 6.97 ± 2.6) or an overall consideration of balance and coordination training as ‘moderate’ intensity exercise (47%, 6.6 ± 2) with reduced overall agreement.)

### Recommendations to improve reporting quality of exercise intervention studies in older adults (> 60 years) and for future meta-analysis processes

Moreover, experts were asked to give recommendations for relevant aspects that will enhance the reporting quality of future interventions studies with older adults. The results are summarized in Table 6:

**Table 6:** Recommendations to improve the reporting quality of exercise intervention studies in older adults (> 60 years)

Suggestions for high-quality reporting of physical intervention studies with older adults Describe:	Rating (M ± SD)
Exercise program modality/FITT principles (type, frequency, duration) (97%)	9.1 ± **1.1**
Exercise progression (94%)	9.1 ± **1.2**
Volume of training (sets, reps, minutes) (94%)	9.1 ± **1.3**
Type of control group (94%)	9.0 ± **1.6**
% of HR max/HR reserve (97%)	8.9 ± **1.1**
The physical fitness of the target group at baseline (97%)	8.8 ± **1.6**
Exercise Intensity (weight, speed, distance) (92%)	8.7 ± **1.4**
Resting Time (92%)	8.7 ± **1.5**
Methods used to assess exercise intensity (89%)	8.7 ± **1.5**
RPE/Fatigue before and after (94%)	8.6 ± **1.5**
Intensity changes in different exercise types (85%)	8.5 ± **1.8**
Adverse events like injuries (89%)	8.3 ± **1.9**
Setting (home based/group) (92%)	8.2 ± **2.0**
METs (89%)	8.1 ± **2.5**
% Maximal oxygen consumption/intensity at blood lactate threshold (83%)	8.0 ± **2.2**
Time of the day (75%)	7.5 ± **2.4**

*Abbreviations*: *HR* Heart rate, *RPE* Rating of perceived exertion, *METs* Metabolic equivalent of task

In addition to these aspects, the experts recommended using accelerometry or other objective measures or engagement (80%; 7.7 ± 2.3) to control intensity.

Finally, the experts gave recommendations for improving the reporting quality as well as additional steps for the conduction of meta-analysis. These results are summarized in Table 7.

**Table 7:** *Recommendations to improve reporting quality of exercise intervention reviews in older adults (*> *60 years)*

Domain	Recommendations and benefits of reporting and analyzing exercise intensity for systematic reviews and meta-analysis (% of Ratings ≥ 7)	Average Rating (M ± SD)
Moderator	The % of subjective or objective maximum (i.e., % of maximum heart rate) should be integrated as a moderator for the meta-analysis. (95%)	8.6 ± **1.2**
	The intensity categories (light, moderate, vigorous, high) should be considered as moderators. (90%)	8.6 ± **1.6**
Effects	Reviews should look for indication that researchers clearly followed best available evidence such as described by American College of Sports Medicine (ACSM) [25], and TIDieR checklist. (90%)	8.5 ± **1.4**
	The physical fitness at baseline should be integrated as a moderator variable. (90%)	8.4 ± **1.6**
	Intensity can be one outcome measure to explore the effect of an intervention. (80%)	8.3 ± **2.2**
Other recommendations	The review authors should reach out to the first author when information (e.g., about intensity) is missing. (78%)	8.2 ± **2.0**
	An intensity score/scale system should be proposed in order to have a semiquantitative description of the different levels of intensity (and their expected effects) e.g., standardized list such as the Compendium of Physical Activities [27]. (85%)	8.2 ± **1.9**
	Studies not reporting intensity levels should also be included in subanalysis (based on frequency, duration, type). (85%)	8.1 ± **2.0**
	All data should be converted to METs for the meta-analysis. (68%)	7.2 ± **2.5**
	A lack of objective measurements to describe exercise intensity should be mentioned in the quality assessment. (89%)	8.5 ± **2.2**
	Trust the authors/suggest accepting if they define an intervention e.g., as moderate. (66%)	7.1 ± **2.4**

*Abbreviations*: *METs* Metabolic equivalent of task

## Discussion

The primary aim of this Delphi process was to consolidate expert knowledge about exercise intensity within training interventions for older adults. This comprises three subgoals: (1) enhance the understanding of the importance of reporting detailed exercise characteristics in the interdisciplinary setting of aging research, (2) support future authors of systematic reviews who aim to summarize the evidence of exercise interventions, and (3) derive optimal practice recommendations for reporting exercise characteristics in future intervention studies.

### Importance of reporting exercise intensity

Within this Delphi process, the experts' opinions on the importance of reporting exercise intensity could be categorized into three domains: (1) deduction, (2) individualization, and (3) safety of the participants.

In terms of deduction and interpretation of study results or potential adaptations, the experts highlighted the importance of training planning and monitoring. These aspects are classical components of training load control (e.g., progression), revealed in a long tradition of training and exercise physiology research [1, 17, 36]. Moreover, the experts claimed the relevance of describing and controlling exercise intensity in terms of goal-directed adaptations [37]. Precise exercise training prescription necessitates considering all training components. For instance, according to one of the most classical aerobic training principles, the duration of exercise training depends on exercise intensity. Higher intensity typically means shorter duration. Similarly, in resistance exercise, it is advised to take at least one day to recover between training days during the weekly microcycle [38]. Therefore, all the components of exercise training should be prescribed with accuracy.

The second category involves tailoring the exercise program to individual needs. Exercise content and intensity should align with the health and fitness levels of older individuals [39].

In addition, this tailoring of loads and doses enhances older individuals´ safety during exercise and might help to avoid adverse events such as overload or exhaustion. Even in general physical activity guidelines that refer to the public, the intensity of the activity is essential and highlighted by scientists. For example, physical activity intensity is one of the parameters that the updated guidelines on physical activity and sedentary behavior of the WHO focused on [40]. For instance, for aerobic-related activities, the guidelines state, ‘Older adults should do at least 150–300 min of moderate-intensity aerobic physical activity; or at least 75–150 min of vigorous-intensity aerobic physical activity; or an equivalent combination of moderate- and vigorous intensity activity throughout the week, for substantial health benefits. Taking all into account, the experts clearly stated that reporting and monitoring exercise intensity in research studies is essential in creating personalized exercise programs for older adults.

Additionally, the experts stated that reporting and analyzing exercise intensity might help prevent potential harm or adverse events. It is of utmost importance that older adults can safely participate in exercise training programs. Even many older adults who have chronic diseases can exercise safely if the training intensity is monitored.

### Expert categorization of exercise intensity

The majority of the experts categorize the types of exercise according to the common recommendations of exercise description for light, moderate, vigorous, and high intensity in the classical literature [27, 28].

However, a divergence arises for yoga in terms of mind–body exercises. While experts in exercise science and exercise physiology categorize yoga as light, other professions rated yoga into the moderate intensity category. One potential explanation might be that the number of different forms of Yoga aggravates the categorization (for a review cf. Cramer et al., 2016 [41]). The different forms of Yoga have some similar and some divergent content. For example, a hatha yoga routine in a study by Clay and colleagues (2005) required 14.5% VO2R, which can be considered a very light intensity and was significantly lighter than 44.8% VO2R for walking at 93.86 m.min(-1) (3.5 mph) [42]. The same classification as light-intensity exercise was reported in a systematic review on yoga [43] as well as for Qigong [44].

Other forms and contents of Yoga (e.g., Asana) require more muscle strength for trunk stabilization as well as muscle flexibility [45]. Indeed, this might be a more demanding form of yoga in comparison to yoga forms that mainly include body awareness and breathing exercises, especially for older adults. It is unclear whether all professions that are designing training interventions for older adults have detailed knowledge about the differences within forms of yoga and how these differences refer to the intensity of a program.

Comparable results were found for balance and coordination training. While the majority of experts categorized balance and coordination training as light intensity, it might be moderate to vigorous if stepping or additional cognitive demand (e.g., dual-tasking) is included. Therefore, it is necessary to briefly define and conduct an exercise program in accordance with the baseline level of each person, the FITT principles and the control of the exercise intensity of an intervention, e.g., by using a BORG scale or heart rate monitoring [26].

There were some conflicting results about the categories light, moderate, vigorous, and high. Some of the participants suggested using only three categories: light, moderate, and merging the vigorous and high categories as one and calling it ‘high’. As mentioned above, the classical recommendation separates high intensities into two categories: a) vigorous and b) high or near maximal to maximal [26, 27]. We followed the classical recommendations for the high intensities and used four categories. The latest separation allows distinguishing studies that use vigorous intensity from those that use very high intensity (i.e., high-intensity interval training). Indeed, high-intensity interval training programs can be considered a safe, well-tolerated, and beneficial exercise form in older adults [46].

The experts´ recommendations for calculating exercise intensity, e.g., for combined exercises or multicomponent programs, again showed some contrary aspects. While the majority of the experts suggested building a weighted average of the components, there was also an enormous agreement to use the single components separately. Within the literature, multicomponent exercise is classified as moderate-intensity exercise for sedentary middle-aged and older adults or those with a maximum exercise capacity of 5–7 METs and is classified as low-intensity exercise for young people [47]. Following these categorizations, building an average might be most suitable; however, both methods seem to be suitable.

Finally, the experts gave some potential advice for calculating exercise intensity if there was a progression within the PE. These comprise, e.g., the delta changes from the original level or use the mean value over the whole time (cf. Table 5). However, to allow these calculations, gold standards for exercise conduction (e.g., following Hecksteden et al., 2018 [17]) and descriptions in interventional studies of older adults are needed.

### Recommendations for reporting and controlling exercise interventions in older adults

In line with the current state of the art, the experts claim to report the exercise characteristics, addressing the FITT principles and additional principles of training control (cf. Table 6). Moreover, the experts mention some important physiological aspects of adaptation mechanisms following PE. These are the physical fitness of the target group at baseline, resting times, perceived exertion or fatigue before and after the PE, or maximal energy consumption of the participants. With respect to this specific exercise physiology knowledge, it would be helpful to create a list of minimal requirements that need to be fulfilled if PE is conducted for older adults. To enhance the effectiveness of exercise interventions, interdisciplinary work in public health should be conducted in collaboration with exercise science or exercise physiology experts.

Moreover, the experts referred to intensity control mechanisms. Exercise intensity can be ruled with various methods [20]. Traditionally, heart rate was the most popular method in terms of exercise intensity prescription of aerobic forms of exercise. In such exercise training forms, exercise intensity usually prescribed as a percentage of maximum heart rate (HRmax) or heart rate reserve (HRR) is commonly used when HR is the parameter we consider for the intensity indicator.

Other objective methods include the percentage of maximal (VO_2_max)/peak oxygen uptake (VO_2_peak) or oxygen consumption reserve (VO_2_R), the intensity at aerobic, anaerobic, and lactate thresholds, and maximal capacity of exercise (i.e., [26]). The assessment of the above parameters requires laboratory assessment (in the case of direct evaluation of such parameters) and may not be suitable for some older individuals. With regard to subjective methods of measuring exercise intensity, the most popular method is using the Borg Rating of Perceived Exertion (RPE) or specific scales for particular diseases. Regarding resistance/strength training, the dominant methods are 1-repetition maximum (1-RM) and RPE [26].

With respect to creating a future meta-analysis, the experts suggest that exercise intensity should be integrated as a control variable or moderator of the effectiveness of an intervention. This will allow us to gain more insights into potential dose‒response relationships of specific PEs in the future.

Finally, the authors strongly agree with the experts´ recommendation to integrate the description of exercise characteristics into the quality assessment of systematic reviews and meta-analyses. Exercise prescription is not solely dependent on intensity, as highlighted in the introduction. It emphasizes that a comprehensive approach is required when determining the appropriate exercise training regimen. However, reporting and understanding the intensity of physical activity poses greater challenges compared to the other components of the FITT principle, particularly for individuals lacking a background in exercise science or exercise physiology. The reporting of the intervention details and following at least the FITT principles are as important as the common criteria of evidence-based medicine, such as the randomization process, to conclude the effectiveness and evidence of a PE intervention because it helps to understand potential adaptation mechanisms that could have been addressed by the PE (cf. also [31]). Moreover, these aspects should also be integrated into the common reporting guidelines for high-quality intervention studies.

### Limitations

In addition to the potential strengths of this Delphi process resulting from the significant number of participating experts, there are also some limitations. First, it must be stated that some of the qualitative answers in the first and second rounds showed some problems with the English language. According to the anonymous nature of the questionnaire, the authors of this Delphi study needed to discuss some of the answers to clarify the potential content. Moreover, the language problems also led to some answers that did not fit the questions; therefore, these comments needed to be deleted. Moreover, many participants were not experts in exercise science or exercise physiology (i.e. nursing, pharmacology, Urban and rural development background etc.; cf. Table 8 in the Annex). Nevertheless, we kept the answers of all participants because we favor the idea of an interdisciplinary understanding and agreement that might be an additional benefit of this Delphi process. Unfortunately, a calculation of the stability of the consensus was not foreseen. Future studies should plan these measurements in advance. Finally, when discussing exercise intensity in older adults, we are assuming a generally healthy population, knowing that a different approach must be made for special populations with performance-limiting pathologies.

## Conclusions

Research on physical exercise is an interdisciplinary field that includes a variety of different areas of public health. This leads to inconsistent reporting of relevant exercise characteristics such as intensity.

This study resulted in achieving consensus on three key aspects:

1. participating experts of this Delphi survey agreed on the importance of reporting exercise intensity for the deduction, individualization, and safety of older participants within an exercise program.
2. Moreover, this Delphi survey revealed expert agreement on categorizing different exercise types for older adults into light, moderate, vigorous, and high intensity.
3. Finally, the survey revealed valuable recommendations for conducting and reporting future exercise interventions for older adults.

In summary, the results of the current survey can be used to classify the intensity of exercise and suggest a practical approach that can be adopted by the scientific community and applied when conducting systematic reviews and meta-analysis articles when vital and objective information regarding exercise intensity is lacking from the original article.

## Data Availability

All relevant data are within the study, and raw data are available on request by the corresponding author.

## Appendices

**Table 8:** Distribution of professions

Sports and Exercise Sciences (*n* = 15)	Physiotherapy and Rehabilitation (*n* = 7)	Medicine (*n* = 7)	Other (*n* = 4)
- Exercise sciences, exercise physiology (*n* = 3) - Sports Science (*n* = 2) - Exercise and Sport Sciences - Movement sciences, aging, health, Exercise - Physical education, Sport Sciences - Psychology, Training science - Biomechanics, Movement analysis, and performance analysis of sports - Sports, Exercise and Human Performance - Clinical Exercise Physiology - Exercise psychology (*n* = 3)	- Physiotherapy, Rehabilitation (*n* = 2) - Physiotherapy, exercise, orthopedic rehabilitation - Physical therapy - Fall prevention - Geriatric physical therapy - integrated physiology of exercise and metabolism - Mobility, epidemiology, aging, walking, activity	- Sports Medicine - Sports Medicine, Dietetics - Medical Rehabilitation - Pharmacotherapy - Cardiovascular physiology - Preventive Cardiology - Orthopedic surgeon	- Public Health - Urban and rural development - Health care - Healthy aging
