# Correction: Defining and reporting exercise intensity in interventions for older adults: a modified Delphi process

**DOI:** 10.1186/s11556-024-00346-7

**Published:** 2024-05-16

**Authors:** Bettina Wollesen, Mona Herden, Nicola Lamberti, Christoforos D. Giannaki

**Affiliations:** 1 Institute of Human Movement Science, University of Hamburg, Hamburg, Germany; 2 Department of Neuroscience and Rehabilitation, University of Ferrara, Ferrara, Italy; 3 Department of Life Sciences, School of Life and Health Sciences, University of Nicosia, Nicosia, Cyprus

**Correction:** ***Eur Rev Aging Phys Act*** **21, 3 (2024)**

10.1186/s11556-024-00337-8

Following publication of the original article [1], the authors inserted the missing reference labeled as reference [32] in the text on page 3 last paragraph.

Below is the reference details:

Slade, S. C., Dionne, C. E., Underwood, M., Buchbinder, R., Beck, B., Bennell, K., … & White, C. (2016). Consensus on exercise reporting template (CERT): modified Delphi study. Physical therapy, 96(10), 1514–1524.

The paragraph should have read:

A standardized checklist consisting of 16 items was formulated in a previous Delphi study to ensure accurate reporting of exercise programs in clinical trials. However, it should be noted that this checklist was not specifically tailored for older adults [31]. Moreover, this checklist was provided to secure high-quality reporting of future RCTs. Specific recommendations on how to deal with missing reporting of intensity for ongoing meta-analysis was missing in this Delphi process **[32]**.

All other references have been renumbered accordingly.

The original article [1] has been updated.
